# Supplementary material for: scDrugAtlas: an integrative single-cell drug response database for dissecting tumour heterogeneity in therapeutic efficacy
Source: Database (Oxford). 2026 Feb 20;2026:baag010. doi: 10.1093/database/baag010 (PMC12923164; doi:10.1093/database/baag010)
Supplement: baag010_Supplemental_Files [file baag010_supplemental_files.zip › Supplementary Table 1.docx]

**Supplementary Table 1** Detailed hyperparameter settings for machine learning classifiers used in the case studies.

| **Model** | **Case Study 1 (Erlotinib-treated PC9)** | **Case Study 2 (Cetuximab-treated HNSCC)** |
| --- | --- | --- |
| **Random Forest (RF)** | n_estimators=300  max_depth=20  min_samples_split=5  min_samples_leaf=2  max_features='sqrt' | n_estimators=200  max_depth=None  min_samples_split=2  min_samples_leaf=1 |
| **Support Vector Classifier (SVC)** | kernel='rbf'  C=1 | kernel='rbf'  C=10 |
| **Logistic Regression (LR)** | penalty='l2'  C=1 | penalty='l2'  C=0.1 |
| **Multi-layer Perceptron (MLP)** | hidden_layer_sizes=(100,)  alpha=0.01 | hidden_layer_sizes=(100,)  alpha=0.01 |
| **Gaussian Naive Bayes** | Default parameters | Default parameters |
