# Supplementary figures and images for: scDrugAtlas: an integrative single-cell drug response database for dissecting tumour heterogeneity in therapeutic efficacy
Source: Database (Oxford). 2026 Feb 20;2026:baag010. doi: 10.1093/database/baag010 (PMC12923164; doi:10.1093/database/baag010)

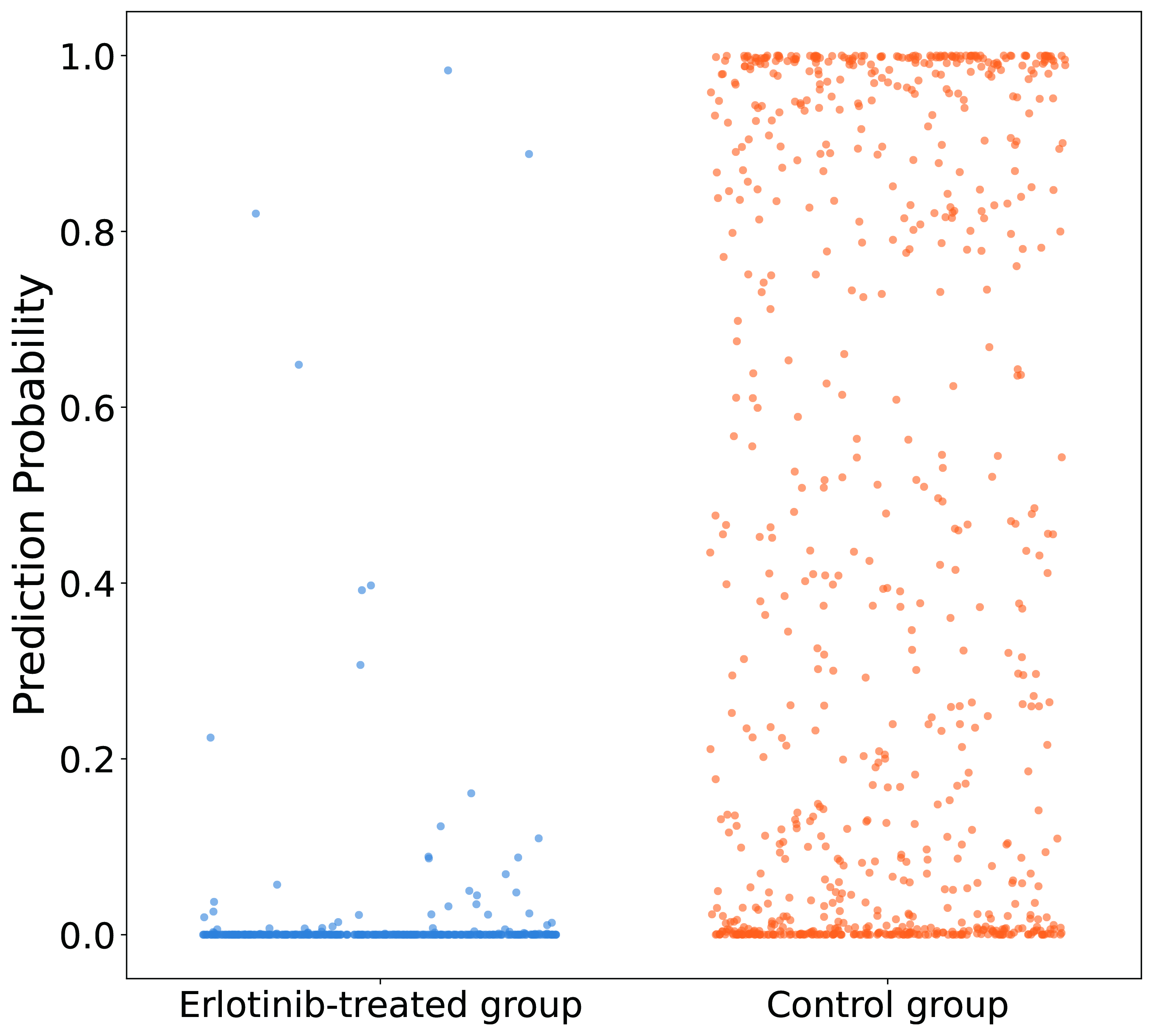

Supplement: baag010_Supplemental_Files [file baag010_supplemental_files.zip › Supplementary Figure 1.tif]

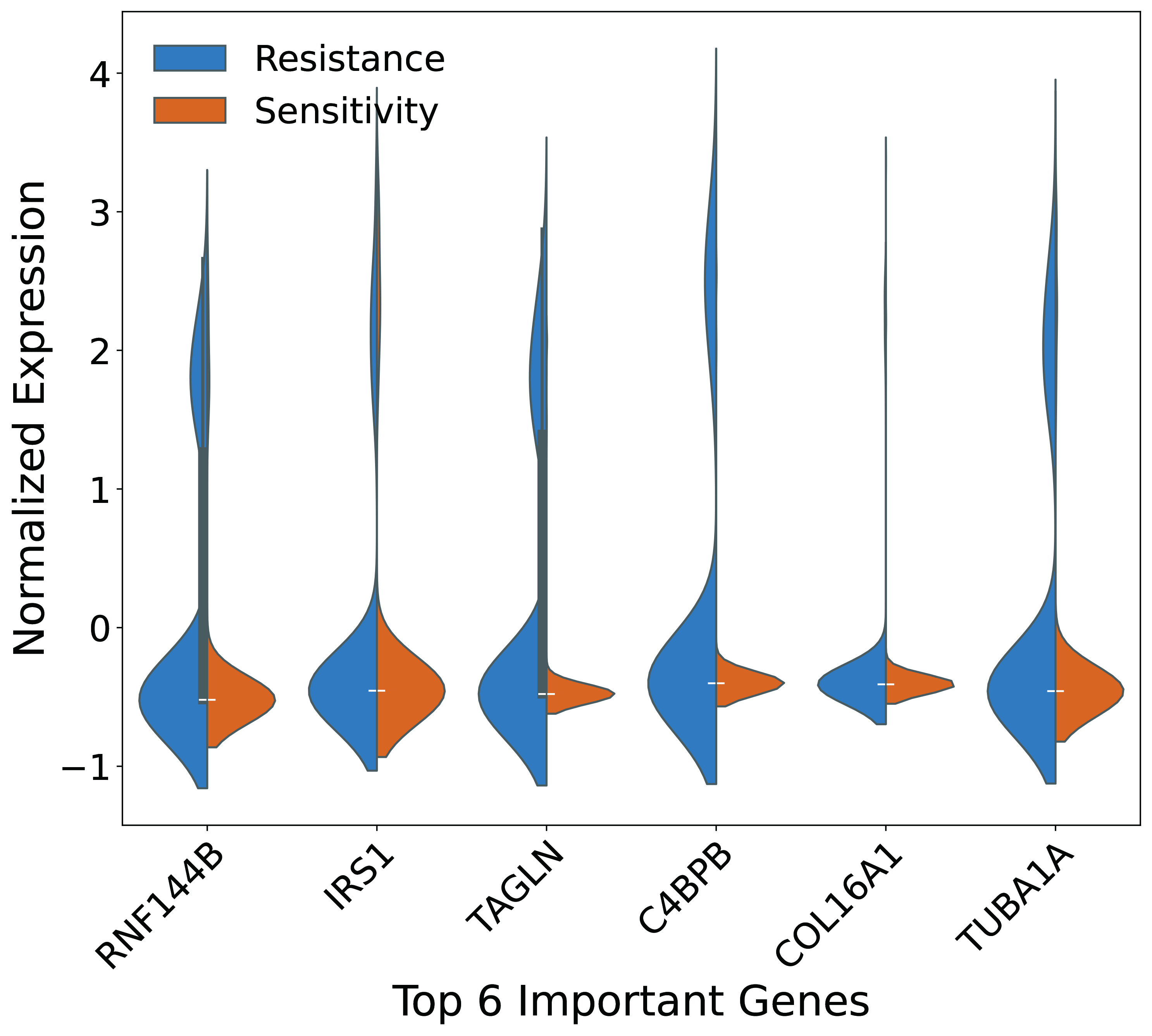

Supplement: baag010_Supplemental_Files [file baag010_supplemental_files.zip › Supplementary Figure 2.tif]

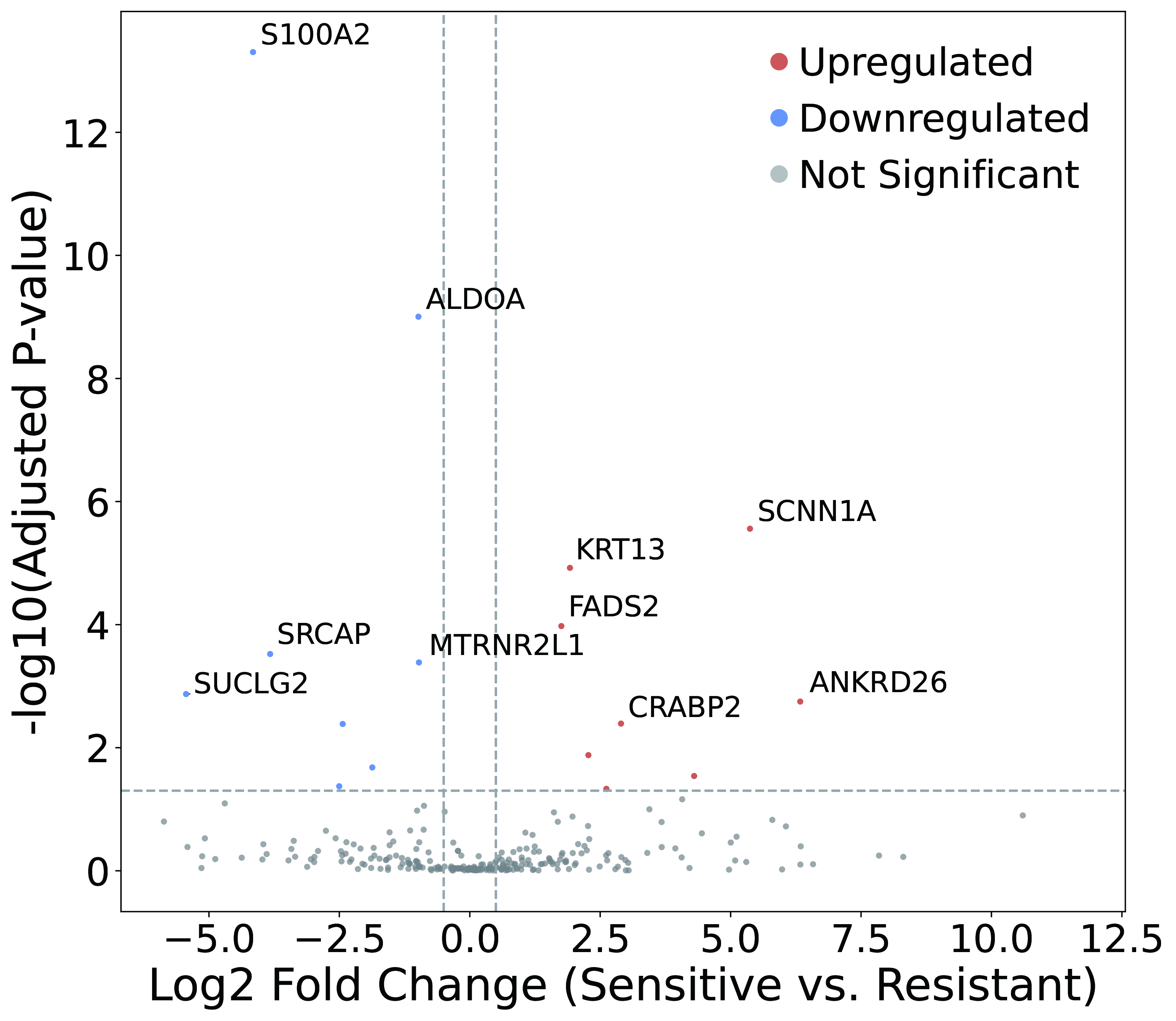

Supplement: baag010_Supplemental_Files [file baag010_supplemental_files.zip › Supplementary Figure 3.tif]

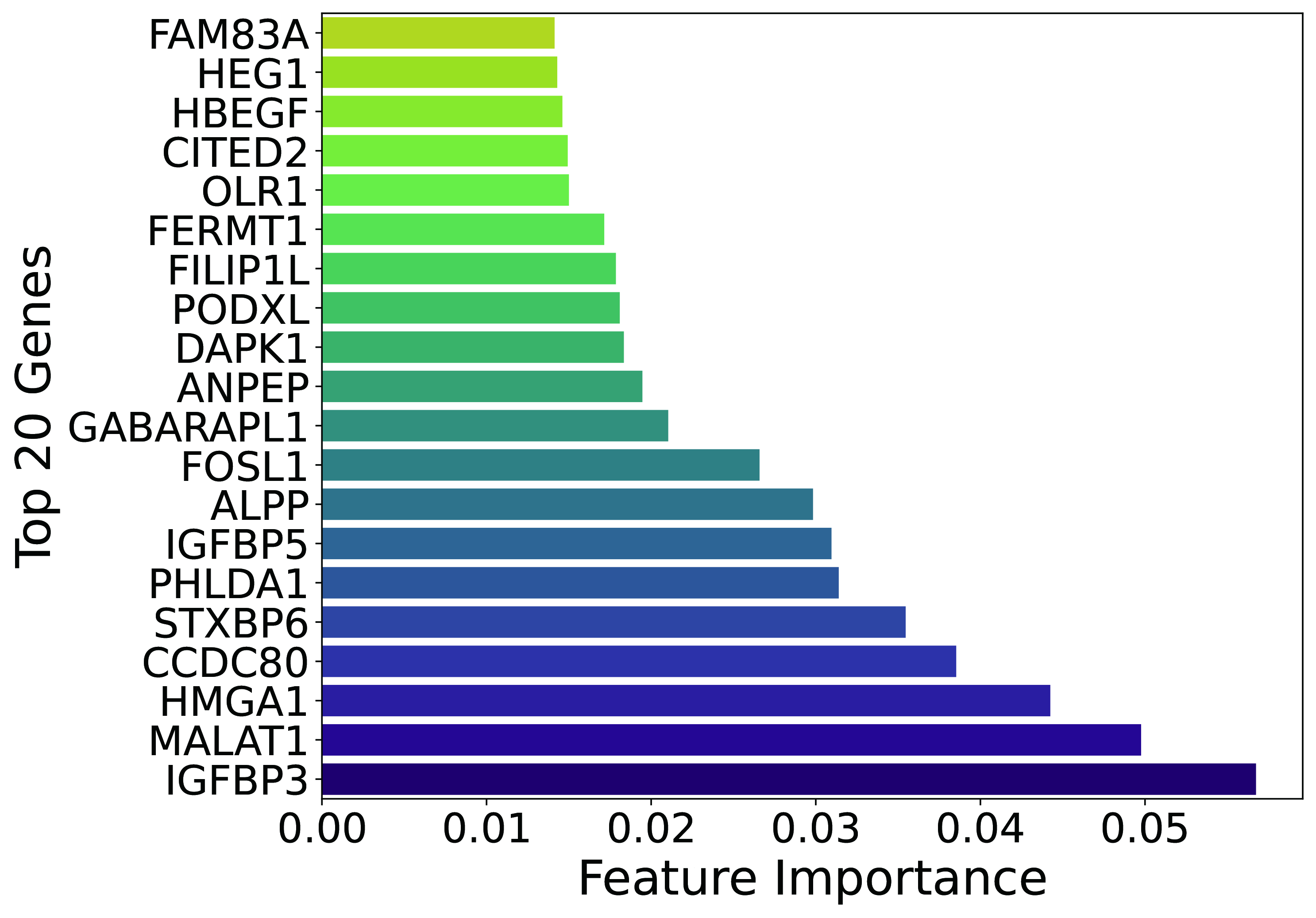

Supplement: baag010_Supplemental_Files [file baag010_supplemental_files.zip › Supplementary Figure 4.tif]

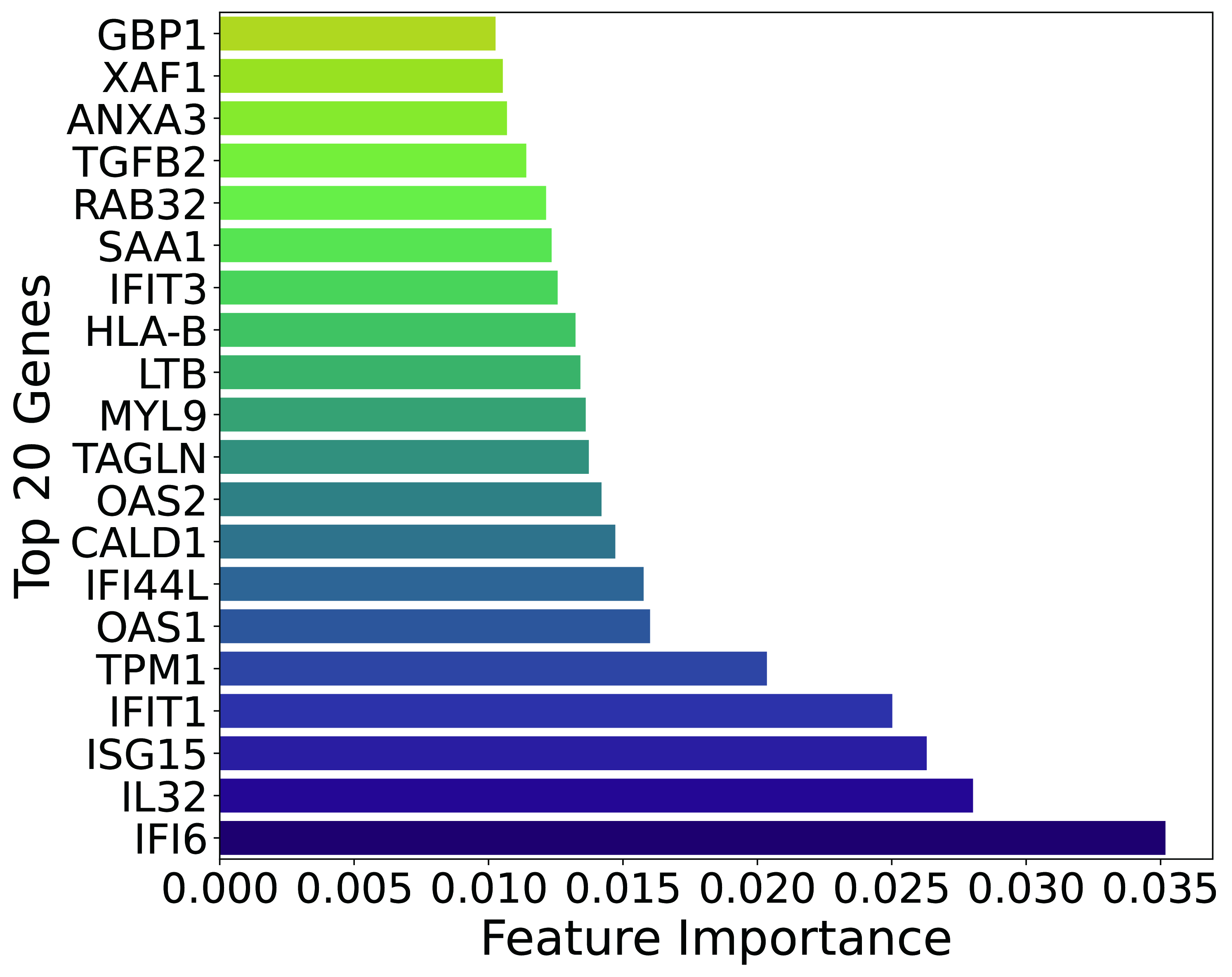

Supplement: baag010_Supplemental_Files [file baag010_supplemental_files.zip › Supplementary Figure 5.tif]
